# Dermal fibroblasts cultured from aged human skin display a pro‐inflammatory phenotype

**DOI:** 10.1002/ccs3.70117

**Published:** 2026-10-06

**Authors:** Yilei Cui, Mai Shi, Christal Worthen, Sherilyn Grill, Jayakrishnan Nandakumar, Yi Zhao, Gary J. Fisher

**Affiliations:** ^1^ Department of Dermatology University of Michigan Medical School Ann Arbor Michigan USA; ^2^ Department of Dermatology Beijing Tsinghua Changgung Hospital Beijing China; ^3^ Molecular, Cellular, and Developmental Biology University of Michigan Ann Arbor Michigan USA

**Keywords:** aging, cytokine signaling, dermal fibroblasts, extracellular matrix, extracellular microenvironment, immune activation, pro‐inflammatory phenotype, transcriptomics

## Abstract

Dermal fibroblasts synthesize, organize, and reside within the dermal extracellular matrix (ECM), which provides mechanical support to the skin and a microenvironment critical for dermal cell function. During aging, the ECM becomes fragmented, a change associated with clinical manifestations such as thin, fragile skin and altered fibroblast function, including reduced expression of ECM structural genes and increased expression of pro‐inflammatory mediators. We investigated whether functional alterations of fibroblasts in aged skin reflect adaptation to the aged dermal ECM microenvironment or intrinsic aging responses. Primary dermal fibroblast cultures were established from young (20–30 years; 6 females, 6 males) and aged (>80 years; 6 females, 6 males) sun‐protected skin. No differences in early growth kinetics were observed between groups; however, fibroblasts from aged individuals grew more slowly after approximately 25 population doublings and had a 20% lower final population doubling level. Prior to passage, after brief primary culture (6–8 days), the average telomere length of fibroblasts from aged donors was reduced by approximately 1000 base pairs (∼1 kb). Global gene expression revealed 477 differentially expressed genes (DEGs; padj ≤0.05, absolute log2FC ≥ 0.58), comprising 300 upregulated and 177 downregulated transcripts. Upregulated DEGs and associated protein–protein interaction networks were enriched in cytokine‐ and chemokine‐mediated signaling pathways (*p* < 10^−5^). Downregulated DEGs were enriched in lipid biosynthesis and fatty acid metabolism. Notably, fibroblasts from young and aged skin expressed similar levels of genes encoding ECM structural proteins, including collagens (42 genes), proteoglycans (29 genes), and glycoproteins (157 genes). These findings indicate that fibroblasts from aged skin retain a pro‐inflammatory transcriptional phenotype in primary culture, resembling their phenotype in vivo. In contrast, primary fibroblasts from aged skin express youthful levels of ECM structural genes in primary culture, suggesting that reduced ECM gene expression in vivo reflects dynamic adaptation to the aged dermal microenvironment rather than permanent reduction of synthetic capacity.

## Graphical abstract

Cell‐Autonomous and Microenvironment‐Driven Changes in Human Skin Dermal Fibroblasts During Aging. Aging induces a cell‐autonomous pro‐inflammatory fibroblast phenotype (independent of the ECM microenvironment), with upregulation of 300 genes primarily associated with cytokine and chemokine pathways (e.g., TNF) and downregulation of 177 genes involved in lipid biosynthesis and fatty acid metabolism. In contrast, age‐related dysregulation of fibroblasts' ECM homeostasis, that is, increased degradation and reduced production, is a cell‐extrinsic adaptation to the dermal ECM microenvironment in aged skin.

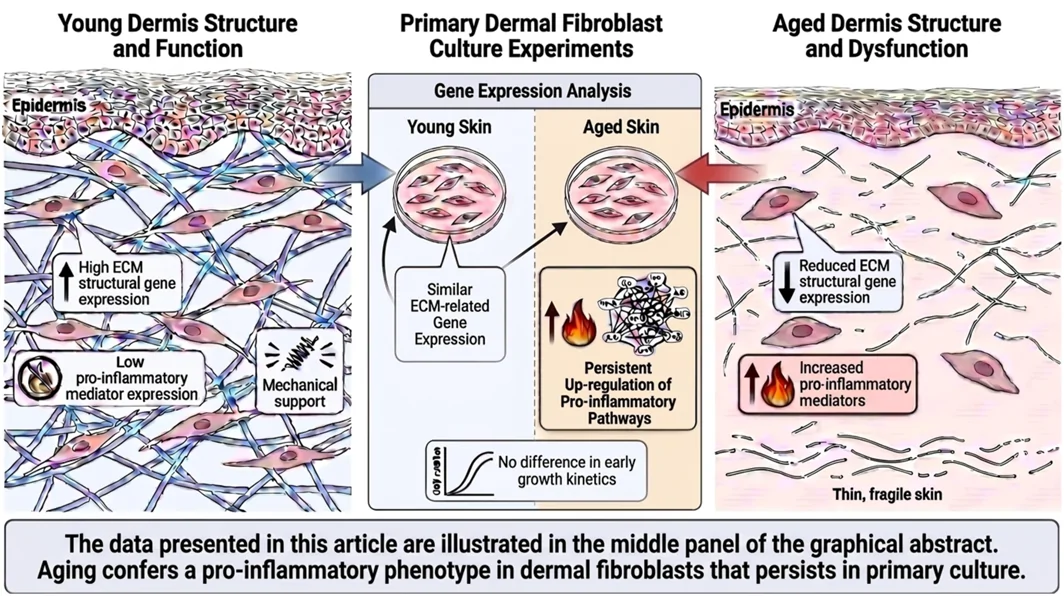

## 1 INTRODUCTION

Skin is the largest organ of the human body, weighing approximately 3.6 kg and covering 1.5–2 m^2^ of the body's surface.^1^ It is an active physical and chemical barrier that protects against infection and dehydration, regulates body temperature, provides immune surveillance, produces hormones, and enables tactile sensation.^2^ In addition to its physiological roles, the skin is the most visible indicator of aging. A youthful appearance is associated with longer life expectancy, whereas aged skin is associated with a shorter life expectancy.^3^ Aging skin is characterized by thinning, sagging, wrinkling, dyspigmentation, and visible vascular changes, reflecting a progressive loss of structural integrity. Functionally, aging skin is associated with increased organismal fragility, impaired wound healing, and greater susceptibility to disease. Thus, age‐related changes in skin structure and function reflect broader declines in tissue resilience across the organism.

Skin consists of three primary layers: the epidermis, dermis, and hypodermis. The epidermis is a stratified squamous epithelium composed primarily of keratinocytes that undergo a highly regulated process of proliferation, differentiation, and terminal cornification to form the stratum corneum, the outermost protective barrier.^4^ Beneath the epidermis lies the dermis, the largest skin compartment by mass and the principal determinant of the skin's mechanical strength and structural integrity. The dermis is composed largely of extracellular matrix (ECM), which is synthesized and maintained by dermal fibroblasts. The hypodermis, the deepest layer, contains adipose tissue, vasculature, sensory neurons, and hair follicles, anchoring the skin to the underlying fascia.

The dermal ECM is composed primarily of structural proteins, including collagens, elastin, proteoglycans, and glycoproteins, which together provide mechanical support and regulate tissue function. Dermal fibroblasts interact with these components through cell‐surface receptors and respond to both biochemical signals in the interstitial fluid and mechanical cues from the ECM. These interactions create a dynamic mechanobiological system that continuously adapts to changes in the dermal microenvironment.^5^, ^6^

Aging of the dermis involves coordinated changes in both the ECM and dermal fibroblasts. There is an intricate interplay of aging among these components within the dermal microenvironment. The aging ECM exhibits progressive loss and fragmentation of structural proteins and increasing disorganization of collagen bundles.^7^, ^8^, ^9^ In parallel, dermal fibroblasts display several cellular hallmarks of aging,^10^ including telomere attrition, reduced biosynthetic activity, and mitochondrial dysfunction associated with increased oxidative stress. However, the extent to which cellular changes arise from intrinsic aging processes versus alterations in the surrounding ECM microenvironment remains unclear.^11^

Morphologically, fibroblasts in young skin exhibit an elongated spindle‐shaped phenotype and maintain extensive attachment to an organized ECM, whereas fibroblasts in aged skin are typically smaller, more rounded, and associated with fragmented, disorganized collagenous ECM.^9^, ^12^ Fibroblasts within aged dermal microenvironments produce lower levels of ECM structural components, express higher levels of matrix‐degrading proteases, including MMP1 and MMP9, and exhibit elevated oxidative stress.^8^, ^9^, ^11^, ^12^, ^13^, ^14^

Experimental evidence suggests that the ECM microenvironment strongly influences fibroblast phenotype. Fibroblasts from young skin placed into an “aged” fragmented three‐dimensional collagen matrix exhibit increased oxidative stress and inflammatory signaling.^11^ Conversely, fibroblasts from aged skin placed into a structurally intact ECM environment partially regain youthful characteristics, including improved proliferative capacity, enhanced responsiveness to growth factors, and reduced oxidative stress.^15^ Similarly, engineered ECM environments can restore more youthful phenotypes in aged fibroblasts and other cell types.^16^, ^17^ These observations suggest that many features of fibroblast aging may reflect adaptive responses to age‐related ECM degeneration rather than strictly cell‐autonomous processes.^18^

Despite these findings, the intrinsic growth capacity and molecular characteristics of aged dermal fibroblasts when removed from their native aged microenvironment remain incompletely defined. It remains unclear whether fibroblasts isolated from aged skin retain stable age‐associated gene expression patterns or are substantially influenced by the surrounding ECM environment. To address this question, we isolated dermal fibroblasts from full‐thickness skin biopsies obtained from young (20–30 years; *n* = 12) and aged (>80 years; *n* = 12) donors from sun‐protected anatomical sites. Fibroblasts were separated from their native ECM and briefly expanded in primary culture (6–8 days). We then compared their gene expression profiles, telomere lengths, and growth characteristics under the same culture conditions. This approach allows us to determine whether key features of dermal fibroblast aging persist independently of the aged dermal microenvironment.

## 2 METHODS

### 2.1 Sample collection

Skin biopsies were obtained from clinically normal adult Caucasian volunteers with no history of inflammatory skin conditions or chronic diseases such as diabetes. Participants were divided into two age groups: a young group (22–30 years old; mean age 26.1 ± 2.5 years; males *n* = 6, females *n* = 6) and an aged group (>80 years old; mean age 82.2 ± 1.8 years; males *n* = 6, females *n* = 6). Three 4‐mm punch biopsies were collected from sun‐protected regions of the hip/buttock from each subject. Each donor sample was maintained as an independent biological replicate throughout the study. All procedures involving human subjects were approved by the University of Michigan Institutional Review Board, and all participants provided written informed consent prior to enrollment in the study.

### 2.2 Cell culture

Primary human dermal fibroblasts were prepared from punch biopsies as previously described.^19^ Briefly, skin samples were digested with bacterial collagenase (Worthington Biochemical Corporation, Lakewood, NJ, USA) to isolate dermal fibroblasts. Fibroblasts from each donor were cultured and maintained separately throughout the study. Isolated fibroblasts were initially plated at 10,000 cells per 60‐mm culture dish in Dulbecco's Modified Eagle Medium (DMEM; Invitrogen Life Technologies, Carlsbad, CA, USA) supplemented with 10% fetal bovine serum (FBS; Invitrogen Life Technologies) and maintained at 37°C in a humidified incubator with 5% CO_2_. When primary cultures reached approximately 70% confluence (6–8 days of culture), cells were collected and divided for three analyses: RNA isolation for RNA sequencing, telomere length analysis, and growth characterization.

### 2.3 Growth kinetics and population doubling analysis

For growth analyses, fibroblasts were replated and passaged individually at approximately 20% confluence (100,000 cells per 100‐mm dish). Cultures were passaged serially whenever confluence reached ∼70%. Population doubling levels (PDLs) were calculated for each individual fibroblast culture as previously described.^20^ Statistical comparison of final population doubling levels between young and aged donor cultures was performed using the independent samples *t*‐test. Longitudinal growth kinetics across serial passages were evaluated by comparing passage‐specific PDL values using independent samples *t*‐tests at defined passage checkpoints.

### 2.4 RNA isolation and bulk RNA sequencing

RNA isolation was performed as previously described.^21^ Briefly, approximately 500,000 cells per donor culture (harvested at 70% confluence during primary culture, days 6–8) were washed with ice‐cold PBS and lysed directly in RLT Lysis Buffer. On‐column genomic DNA digestion was performed using the RNase‐Free DNase Set (Qiagen, Hilden, Germany) for 15 minutes at room temperature to eliminate residual DNA contamination. Total RNA was eluted in 20 μL of nuclease‐free water. RNA concentration and integrity were assessed using the Agilent 2100 Bioanalyzer. All samples met the quality requirements for library preparation, with RIN values ≥ 7 and ≥ 100 ng total RNA per sample. Bulk RNA‐seq libraries were prepared and sequenced by the University of Michigan School of Medicine Advanced Genomics Core Facility (RRID: SCR_025788) on an Illumina sequencing platform using paired‐end 150 bp reads, with a target sequencing depth of approximately 30 million reads per sample. Raw sequencing data were assessed for quality using FastQC, followed by adapter trimming and quality filtering. High‐quality reads were aligned to the human reference genome (GRCh38/hg38) using STAR, and gene‐level read counts were generated using featureCounts with GENCODE gene annotation.

### 2.5 Telomere length analysis

Telomere restriction fragment length analysis was performed as previously described with minor modifications.^22^ Briefly, genomic DNA from approximately 1 million primary cells (harvested at days 6–8) was isolated using the GenElute Kit (Sigma‐Aldrich, St. Louis, MO, USA). Total DNA (2 μg) was digested with HinfI and RsaI overnight at 37°C. Digested DNA and a Lambda DNA‐HindIII ladder (New England Biolabs, Ipswich, MA, USA) were resolved on a 0.8% agarose/1X TBE gel by electrophoresis at 50 V overnight. Gels were depurinated in 0.25 M HCl for 15 min, denatured in 0.5 M NaOH/1.5 M NaCl for 30 min, and neutralized in 1.5 M NaCl/0.5 M Tris‐HCl (pH 7.5) for 30 min. DNA was capillary‐transferred to Hybond N+ membranes (GE Healthcare) in 10X SSC, UV cross‐linked at 1200 μJ, and hybridized overnight at 55°C with γ‐^32^P‐labeled (TTAGGG)_4_ oligonucleotide probes in Church buffer. Membranes were washed with 2× SSC at 55°C and exposed to phosphorimager screens. Signal intensity was quantified using a Typhoon phosphorimager and ImageQuant software (GE Healthcare). Statistical comparison of mean telomere restriction fragment length between young and aged cohorts was performed using the independent samples *t*‐test.

### 2.6 Differential gene expression and bioinformatic analysis

Differential gene expression (DEG) analysis between fibroblasts from young and aged skin was performed using the DESeq2 R package.^23^ Donor sex was initially evaluated as a potential covariate in the statistical model; because no significant sex‐dependent effects on global expression profiles were detected, the primary differential expression model evaluated age group as the main factor. *p*‐values were adjusted using the Benjamini–Hochberg method to control the false discovery rate. Genes with an adjusted *p*‐value (padj) ≤ 0.05 AND an absolute log_2_ fold change ≥0.58 (corresponding to a ≥1.5‐fold change in expression) were defined as significantly differentially expressed DEGs across all analyses. Hierarchical clustering of DEGs was performed using pheatmap.^24^ Principal component analysis (PCA) was conducted using the prcomp function in R based on variance‐stabilized transcript counts.

### 2.7 Functional enrichment and protein–protein interaction (PPI) analysis

Gene Ontology (GO) biological process, cellular component, and molecular function analyses, alongside Kyoto Encyclopedia of Genes and Genomes (KEGG) pathway enrichment analyses, were performed using the clusterProfiler R package with length bias correction (padj <0.05). PPI networks were constructed using the STRING database (version 11.5; http://string‐db.org/) to identify interactions among proteins encoded by the DEGs^25^, ^26^ with a minimum interaction score threshold of 0.4. Densely connected subclusters were resolved using k‐means clustering. Network topography and key hub genes were identified using the CytoHubba plugin with the Maximal Clique Centrality (MCC) algorithm ^27^ within Cytoscape software (version 3.9.1^28^).

## 3 RESULTS

### 3.1 Growth characteristics of dermal fibroblasts from young and aged human skin

To examine intrinsic differences between fibroblasts from young and aged human skin, independent of their native ECM environments, primary dermal fibroblasts were isolated from 4‐mm punch biopsies obtained from sun‐protected hip/buttock skin of young (20–30 years old; 6 females, 6 males) and aged (>80 years old; 6 females, 6 males) donors. Biopsies were enzymatically dispersed into single cells, counted, and 10,000 cells were plated into 60‐mm tissue culture dishes and maintained under standard monolayer culture conditions. Each donor sample was cultured independently throughout the study.

Cell yield from biopsies was similar between young and aged donors. Initial plating was designated passage 0 (P0), and early cellular growth was assessed by measuring the number of days required for cultures to reach approximately 70% confluence. Fibroblasts from young and aged human skin reached this level of confluence in a similar time frame (7.3 ± 0.5 days vs. 6.3 ± 0.5 days, respectively; Figure 1A; evaluating primary culture duration of 6–8 days). No significant sex‐related differences in early growth rates were observed.

**FIGURE 1:**
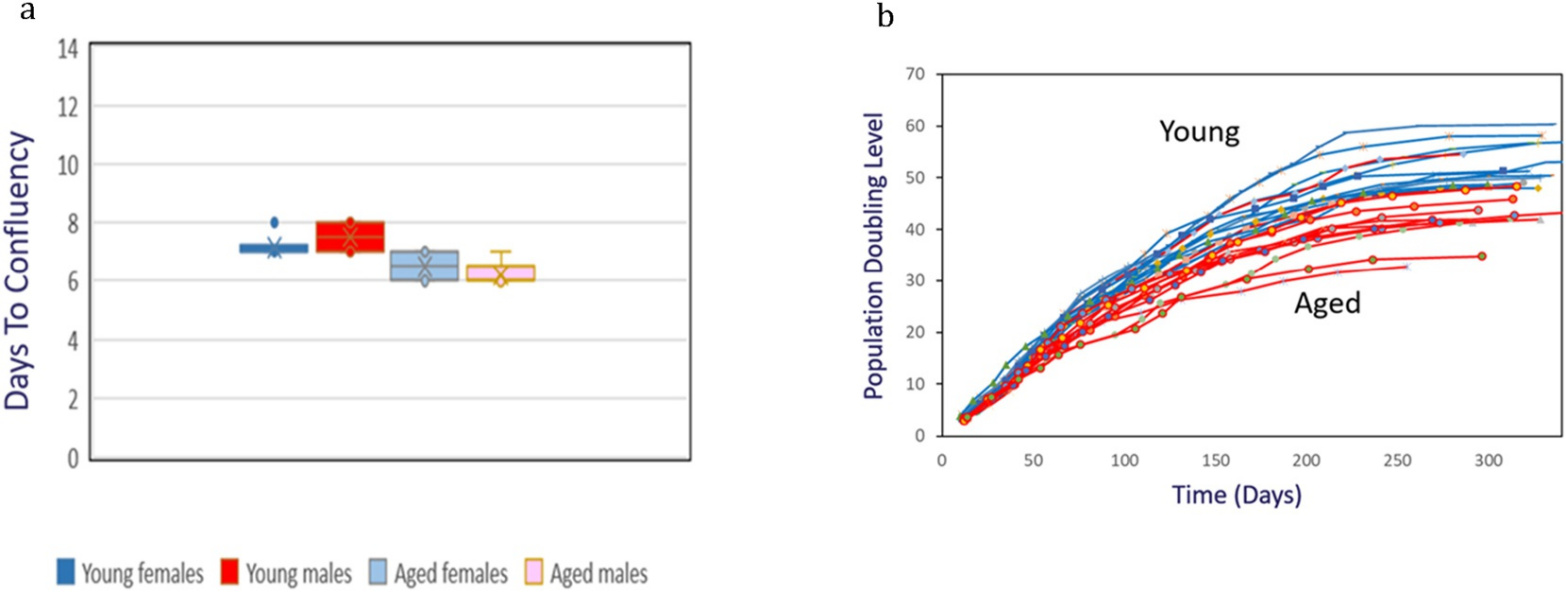
Initial growth kinetics of primary human dermal fibroblasts from young and aged skin are similar in vitro. Primary human dermal fibroblasts from young (20–30 years old; *n* = 12 biological donors) and aged (>80 years old; *n* = 12 biological donors) subjects were harvested from sun‐protected hip/buttock skin, enzymatically dispersed into single cells, and plated at equal density. (A) Initial growth measured as days to reach 70% confluence during primary culture (6–8 days). (B) Long‐term growth kinetics quantified by PDLs across serial passages. Data represent means ± SEM. PDLs, Population doubling levels.

Cells from each donor biopsy were subsequently passaged whenever cultures reached approximately 70% confluence. Cells were enzymatically detached, counted, and replated at 100,000 cells per 100‐mm culture dish for each passage. PDLs were used to compare growth characteristics between young and aged fibroblast cultures over time.^20^ During the first 50 days of culture, fibroblasts from young and aged donors displayed similar growth rates and PDLs (Figure 1B). For example, at passages 2 and 11, the PDL remained indistinguishable between groups (Figure 2A). Differences between the groups became apparent only at later passages (>100 days in culture), when fibroblasts from aged skin exhibited reduced proliferative capacity. By passage 18, these differences were clear (Figure 2A). Overall, the total number of population doublings achieved after harvest was 53 ± 1.8 for fibroblasts derived from young donors and 43 ± 1.5 for fibroblasts derived from aged donors (Figure 2B; independent samples *t*‐test, *p* < 0.001).

**FIGURE 2:**
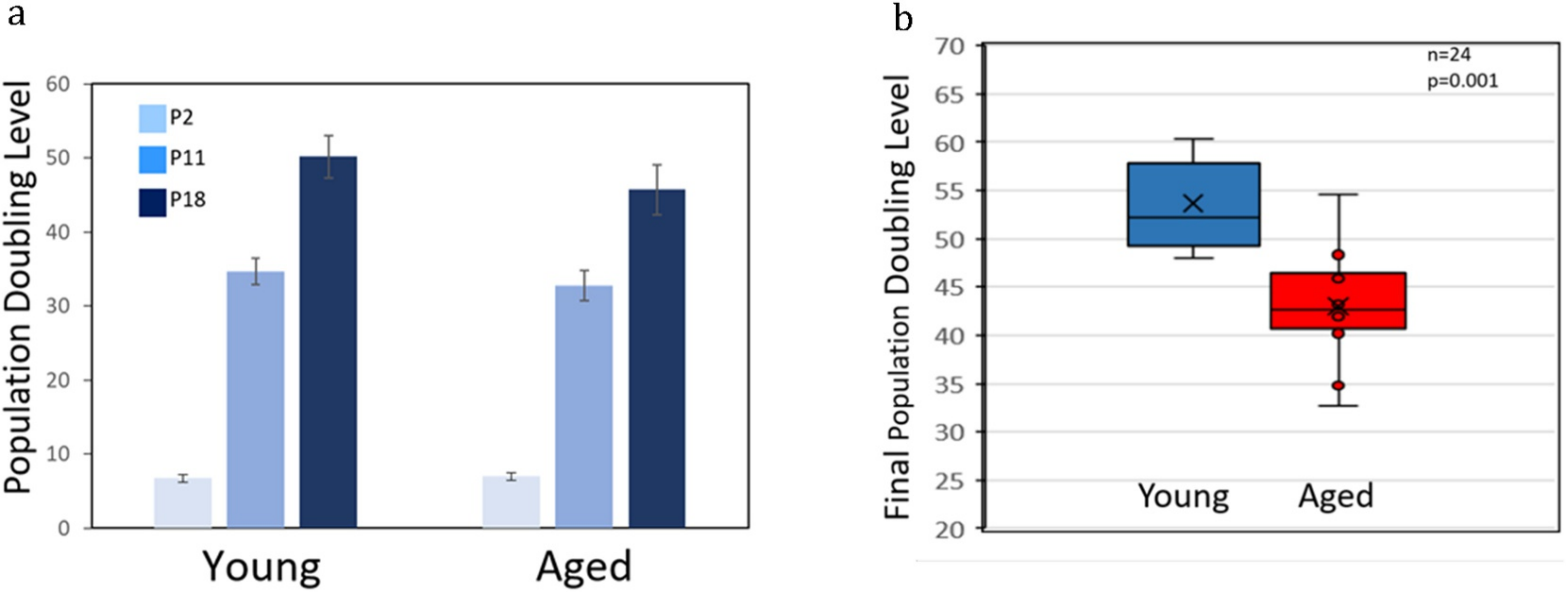
PDLs between young and aged human dermal fibroblasts over serial passage. Primary dermal fibroblasts from young (*n* = 12) and aged (*n* = 12) donors were passaged serially upon reaching 70% confluence. (A) PDLs at indicated passages (P2, P11, P18). (B) Final cumulative PDLs at terminal senescence. Data represent means ± SEM for *n* = 24 total donor samples (12 young donors, 12 aged donors). Statistical comparison was performed using an independent samples *t*‐test (***p < 0.001). PDLs, Population doubling levels.

### 3.2 Telomere length and transcriptomic profiling

To further characterize intrinsic differences between dermal fibroblasts from young and aged human skin, telomere length and gene expression profiles were examined. To minimize potential confounding effects of the donor ECM microenvironment, freshly isolated fibroblasts were briefly cultured under the same conditions until they reached approximately 70% confluence (6–8 days) before analysis.

Changes in telomere length were measured using Southern blot telomere restriction fragment analysis. Telomeres in primary fibroblasts from aged donors were approximately 1000 base pairs (∼1 kb) shorter than those from young donors (mean telomere restriction fragment length of 12 vs. 13 kb, respectively; Figure 3A,B; independent samples *t*‐test, *p* = 0.0016).

**FIGURE 3:**
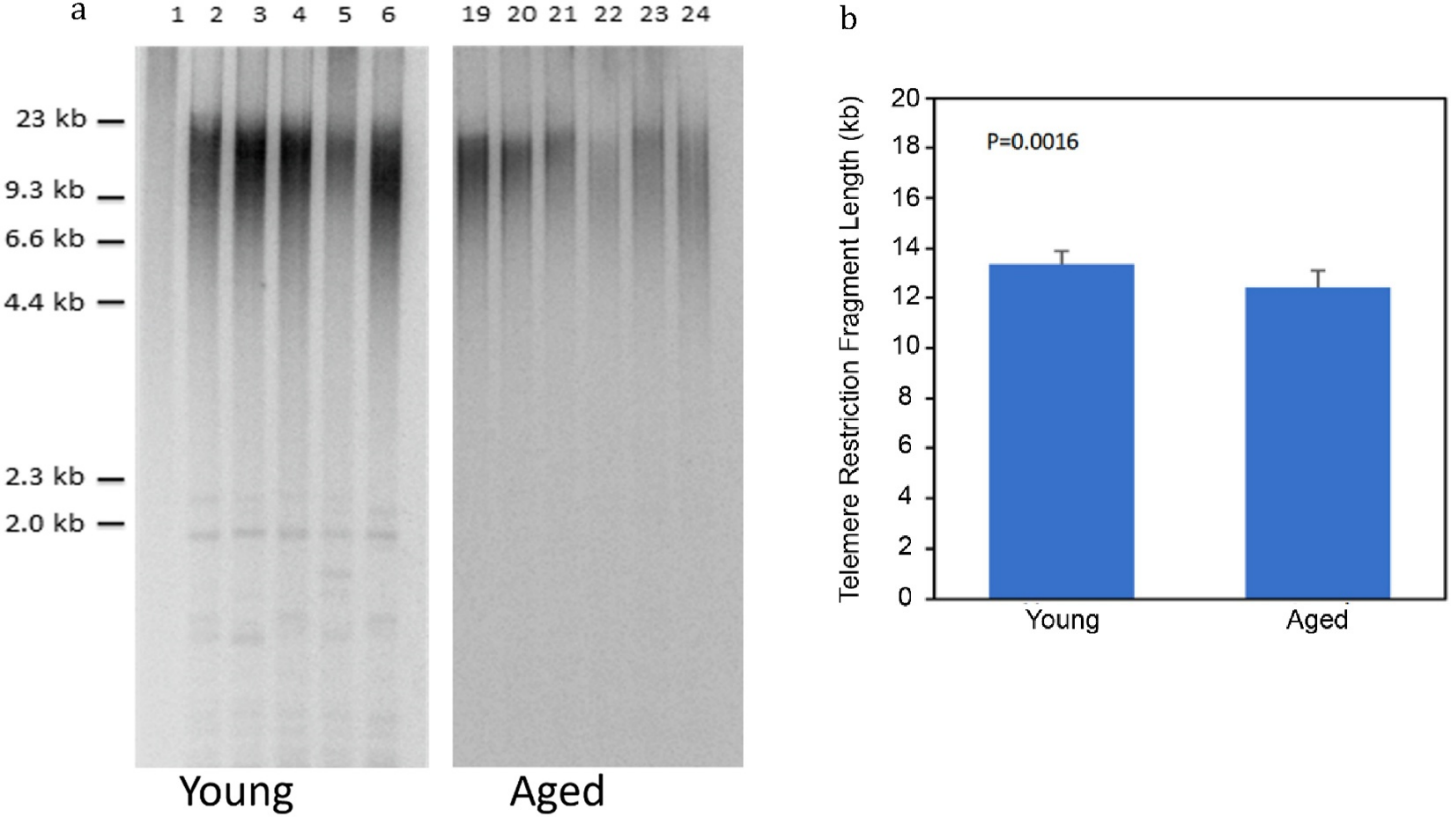
Telomere restriction fragment length analysis of primary dermal fibroblasts. Primary human dermal fibroblasts from young (*n* = 12) and aged (*n* = 12) donors were harvested after brief primary culture (6–8 days). Genomic DNA was digested with HinfI/RsaI and analyzed by Southern blotting. (A) Representative Southern blot image of telomeric DNA fragments. (B) Quantitation of mean telomere restriction fragment lengths. Data are presented as means ± SEM (*n* = 12 biological donors per group). Statistical significance was determined using an independent samples *t*‐test (**p = 0.0016). Fibroblasts from aged donors exhibited an ∼1000 bp (∼1 kb) reduction in mean telomere length compared to young controls. PDLs, Population doubling levels.

Gene expression profiles were then analyzed using global bulk RNA sequencing. After normalization of transcript counts across samples, PCA was performed. The PCA plot revealed an apparent age‐associated separation trend between fibroblasts from aged donors and those from young donors along the primary axes (Figure 4). Fibroblasts from aged donors formed a relatively compact cluster, whereas fibroblasts from young donors displayed a broader distribution, reflecting donor variability. Overlap between individual young and aged donor samples was observed, indicating that while age influences global expression, donor‐to‐donor heterogeneity remains prominent. No distinct clustering based on donor sex was observed.

**FIGURE 4:**
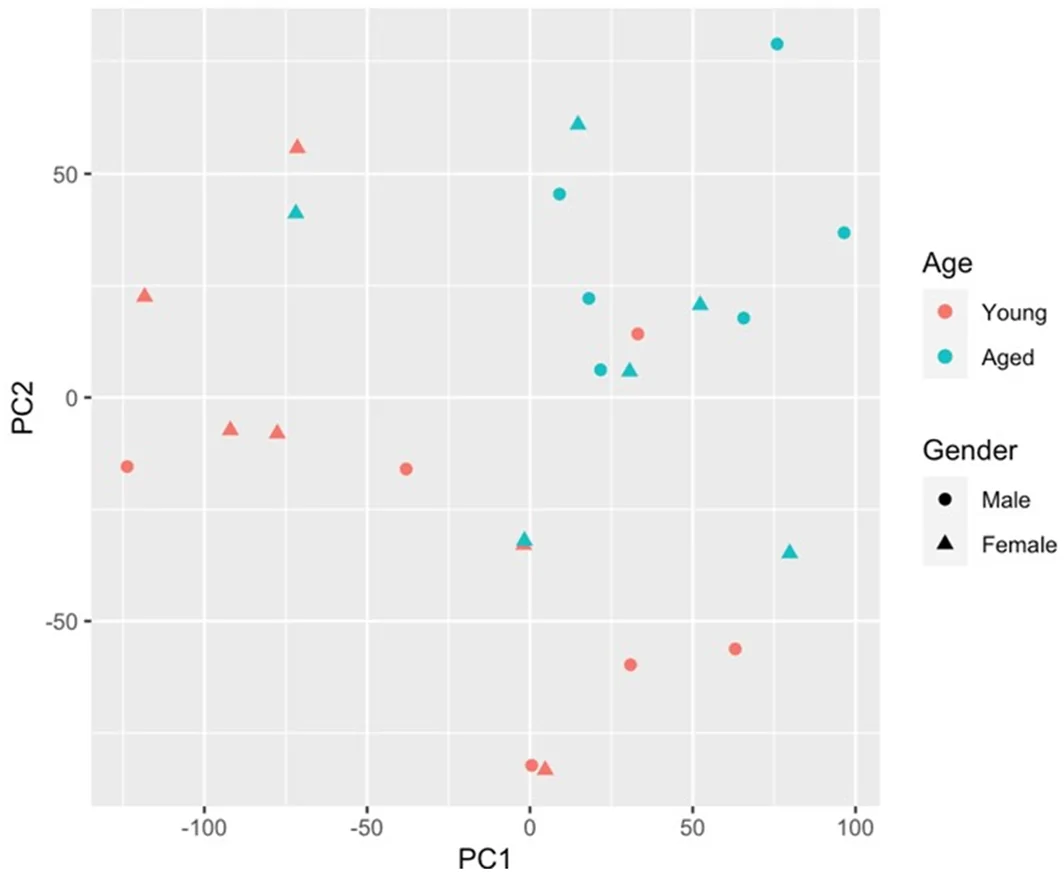
Principal component analysis (PCA) of global transcriptomic data. PCA was performed on variance‐stabilized RNA‐seq transcript counts from primary dermal fibroblasts cultured from young (*n* = 12; blue) and aged (*n* = 12; red) donors. The plot illustrates an apparent age‐associated separation trend along principal components, with individual donor variability and partial group overlap noted. PDLs, Population doubling levels.

Applying standardized differential expression criteria (padj ≤0.05 and absolute log_2_ fold change ≥0.58), a total of 477 genes were identified as DEGs between aged and young fibroblasts. Among these, 300 genes were upregulated and 177 genes were downregulated in fibroblasts from aged donors. A hierarchical clustering heatmap generated from these DEGs demonstrated clear partitioning of transcriptional profiles between the two age groups (Figure 5).

**FIGURE 5:**
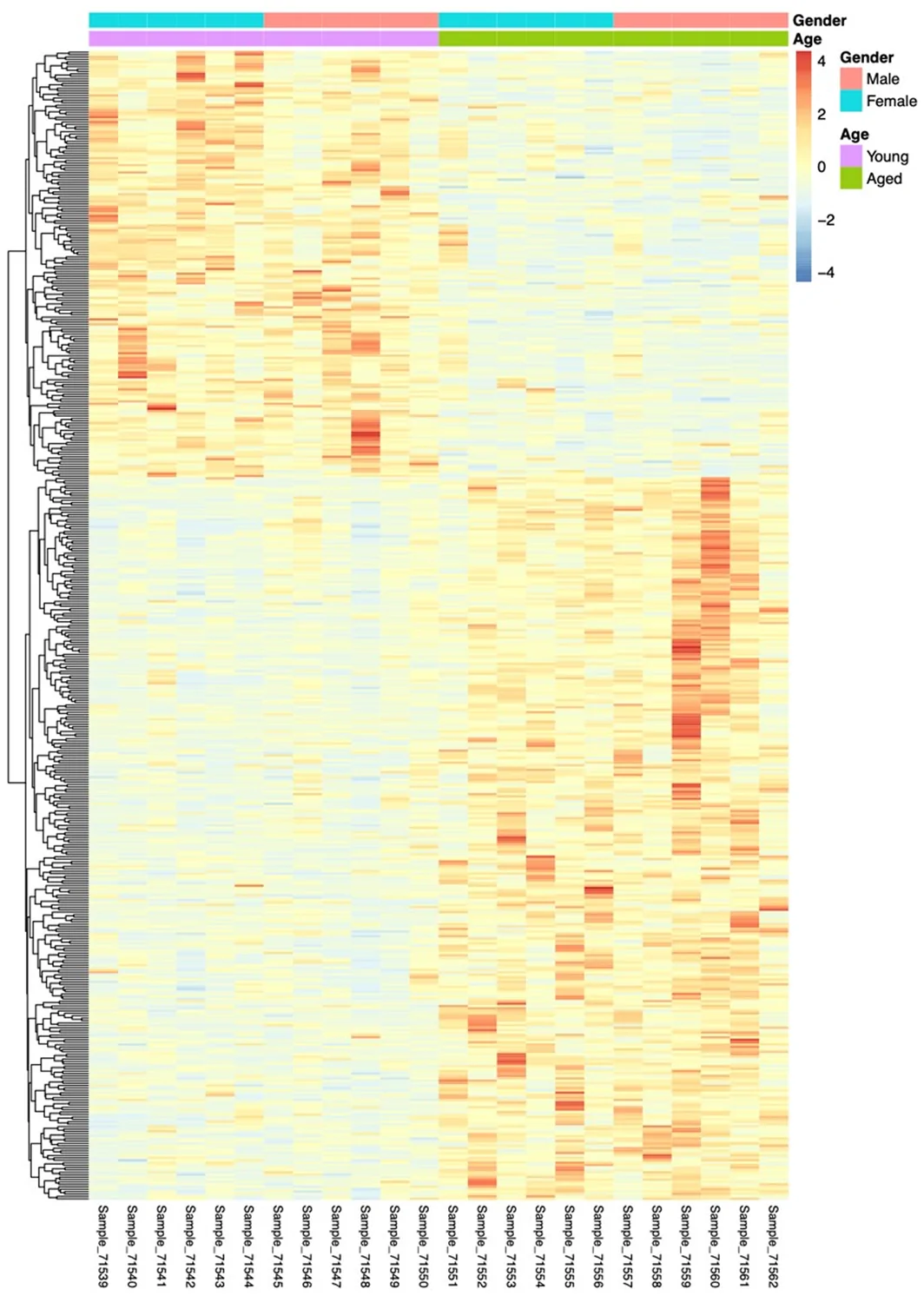
Hierarchical clustering heatmap of DEGs. Global bulk RNA‐seq was performed on primary dermal fibroblasts from young (*n* = 12) and aged (*n* = 12) donors following 6–8 days of culture. DEGs were defined using standardized criteria of an adjusted *p*‐value (padj) ≤ 0.05 AND an absolute log_2_ fold change ≥0.58 (≥1.5‐fold change). Heatmap displays relative expression Z‐scores for 477 DEGs (300 upregulated, 177 downregulated in aged fibroblasts). DEGs, differentially expressed genes.

### 3.3 Matrisome gene expression in primary dermal fibroblasts

Matrisome gene categories defined by Naba et al.^29^ were used to evaluate ECM‐related gene expression. Of the 1026 genes comprising the matrisome database, 732 were detected in our primary fibroblast dataset (228 core matrisome genes and 504 matrisome‐associated genes). Across both young and aged groups, a majority of core ECM structural genes did not significantly differ in expression levels upon primary culture under the same conditions (Figure 6). Specifically, primary fibroblasts from aged donors expressed comparable transcript levels of 42 collagens, 29 proteoglycans, and 157 glycoprotein genes relative to young controls. However, a specific subset of matrisome‐associated genes exhibited differential expression, with aged fibroblasts displaying elevated baseline expression of specific matrix remodeling enzymes and inflammatory matrisome components compared to young cells.

**FIGURE 6:**
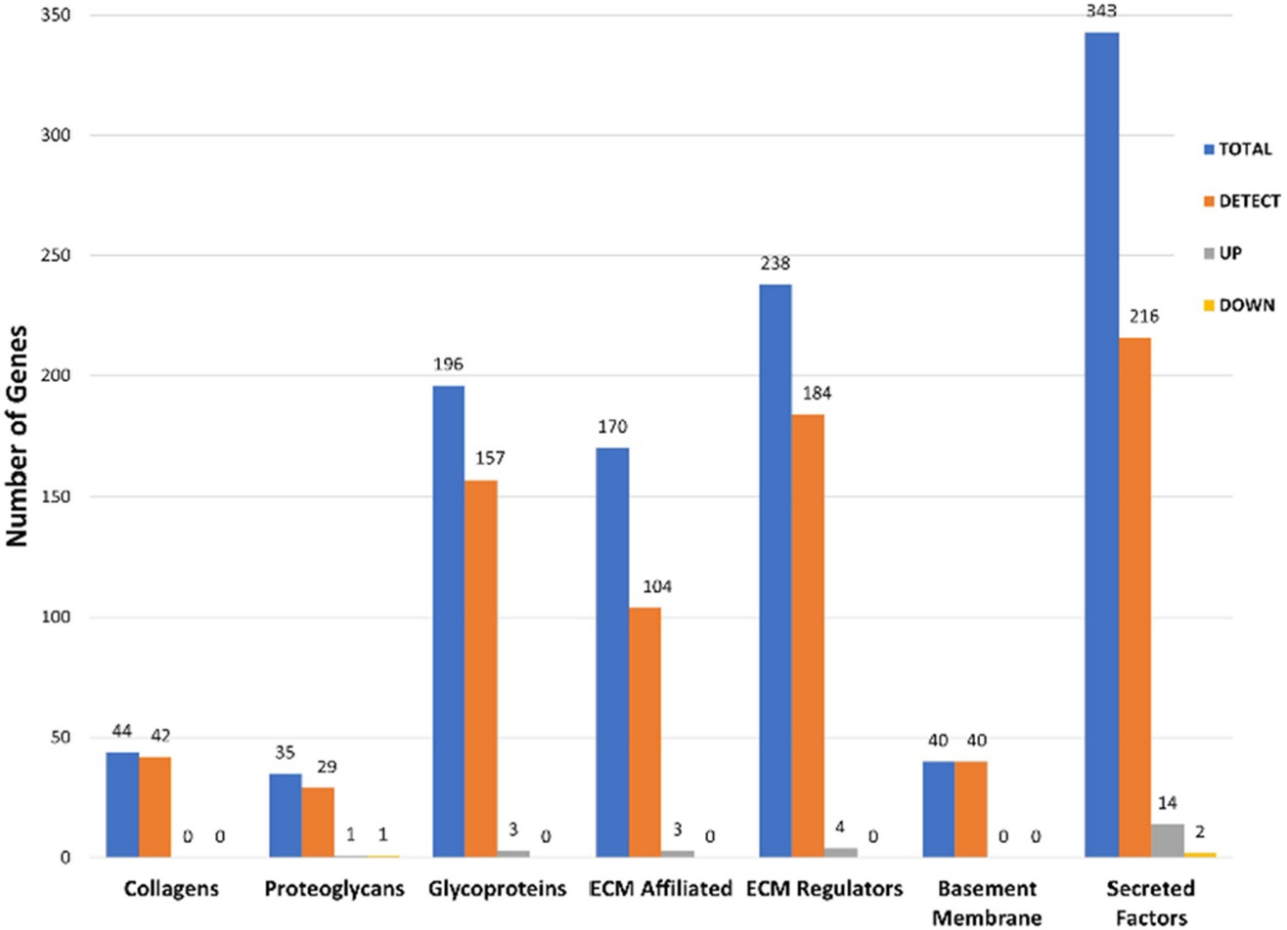
A majority of core ECM structural genes did not significantly differ in primary dermal fibroblast cultures from young and aged human skin. Comparative expression analysis of matrisome gene categories ^29^ between young (*n* = 12) and aged (*n* = 12) primary dermal fibroblasts. TOTAL, number of genes in reference category; DETECT, genes detected in dataset; UP: genes upregulated in aged fibroblasts (padj ≤0.05, log_2_FC ≥ 0.58); DOWN, genes downregulated in aged fibroblasts (padj ≤0.05, log_2_FC ≤ −0.58). Core structural ECM genes (collagens, proteoglycans, glycoproteins) exhibited broadly comparable expression levels across age groups.

### 3.4 Functional enrichment and consolidated PPI network hub analysis

Functional enrichment analysis revealed distinct biological processes enriched among age‐regulated transcripts. Upregulated DEGs in aged fibroblasts were strongly enriched for immune‐ and inflammatory‐related pathways, including cytokine‐mediated signaling (GO biological process), cellular response to cytokines, cytokine–cytokine receptor interaction (KEGG), and TNF signaling (Figure 7A,B). Downregulated DEGs in fibroblasts from aged skin were primarily enriched in lipid metabolism, cholesterol biosynthesis, and fatty acid metabolic pathways (Figure 8A,B).

**FIGURE 7:**
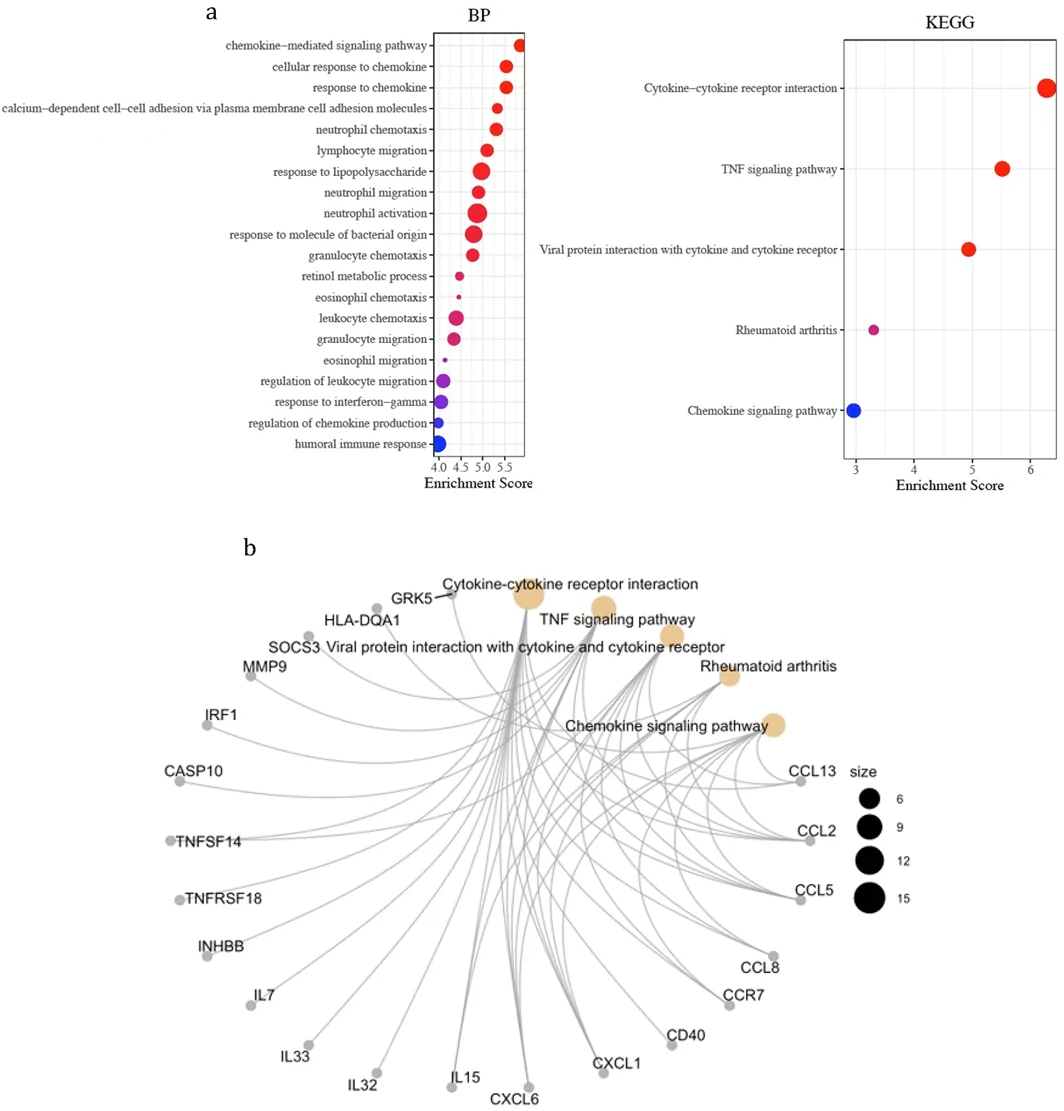
Functional enrichment analysis of upregulated genes in aged dermal fibroblasts. (A) Gene Ontology (GO) biological process and KEGG pathway enrichment analyses for 300 upregulated DEGs in aged fibroblasts (padj <0.05). (B) Gene‐concept network illustrating key genes contributing to top enriched cytokine and chemokine signaling pathways. KEGG, Kyoto Encyclopedia of Genes and Genomes.

**FIGURE 8:**
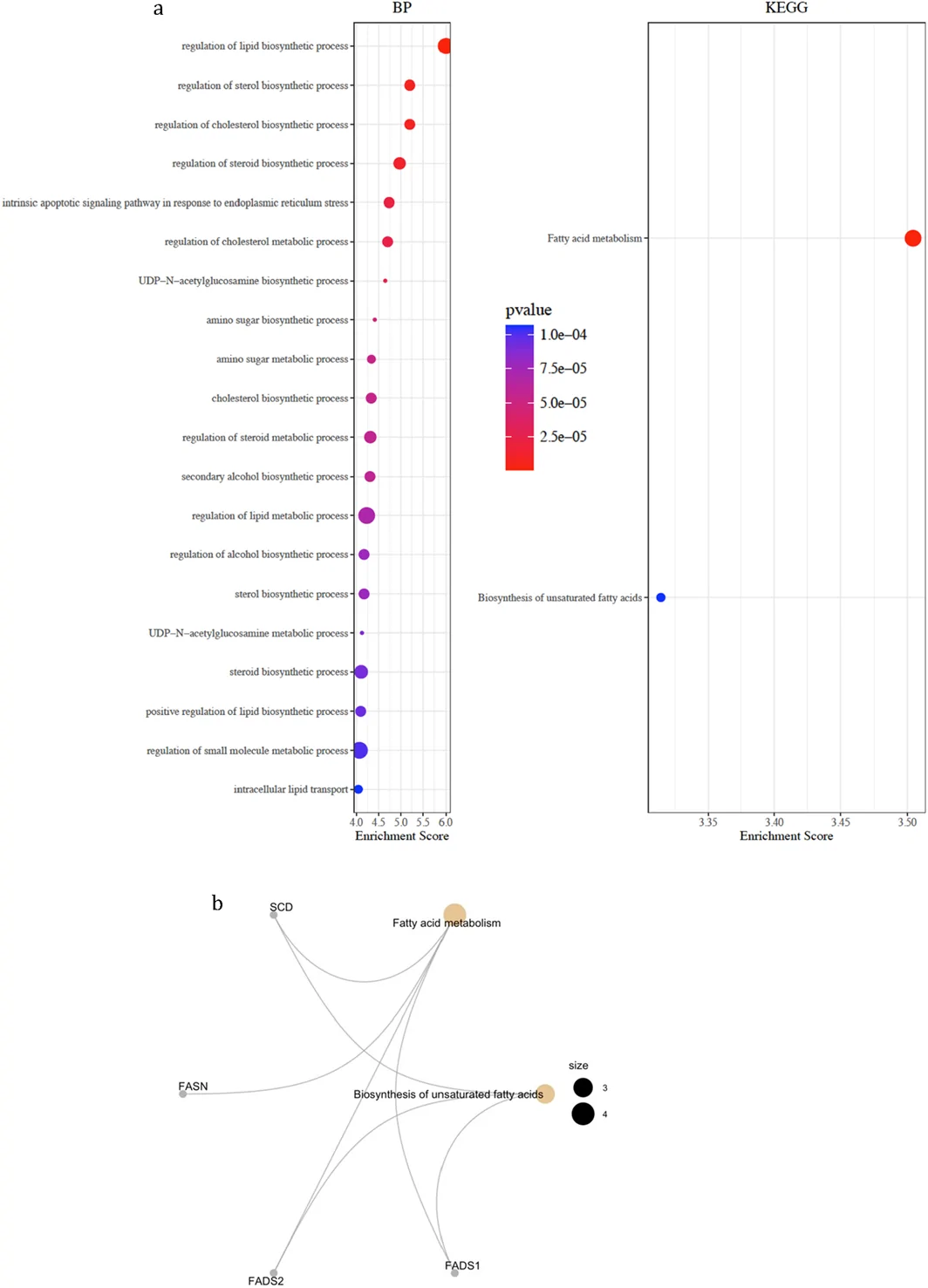
Functional enrichment analysis of downregulated genes in aged dermal fibroblasts. (A) GO biological process and KEGG pathway enrichment analyses for 177 downregulated DEGs in aged fibroblasts (padj <0.05). (B) Gene‐concept network highlighting genes involved in lipid metabolism and cholesterol biosynthesis. KEGG, Kyoto Encyclopedia of Genes and Genomes.

PPI network analysis of the 300 upregulated DEGs via STRING database (Search Tool for the Retrieval of Interacting Genes/Proteins^30^, ^31^) identified a highly interconnected network containing a major k‐means cluster of 90 genes (30% of total upregulated DEGs; Figure 9). Topological analysis using Cytoscape (https://cytoscape.org/) and CytoHubba (MCC algorithm) identified the top‐scoring hub genes stabilizing this pro‐inflammatory network as CCL5, CCL2, CXCL1, CD40, IL15, and MMP9. Pathways most strongly associated with the central hub gene CCL5 included cytokine receptor interactions, chemokine signaling, and TNF‐mediated pathways.

**FIGURE 9:**
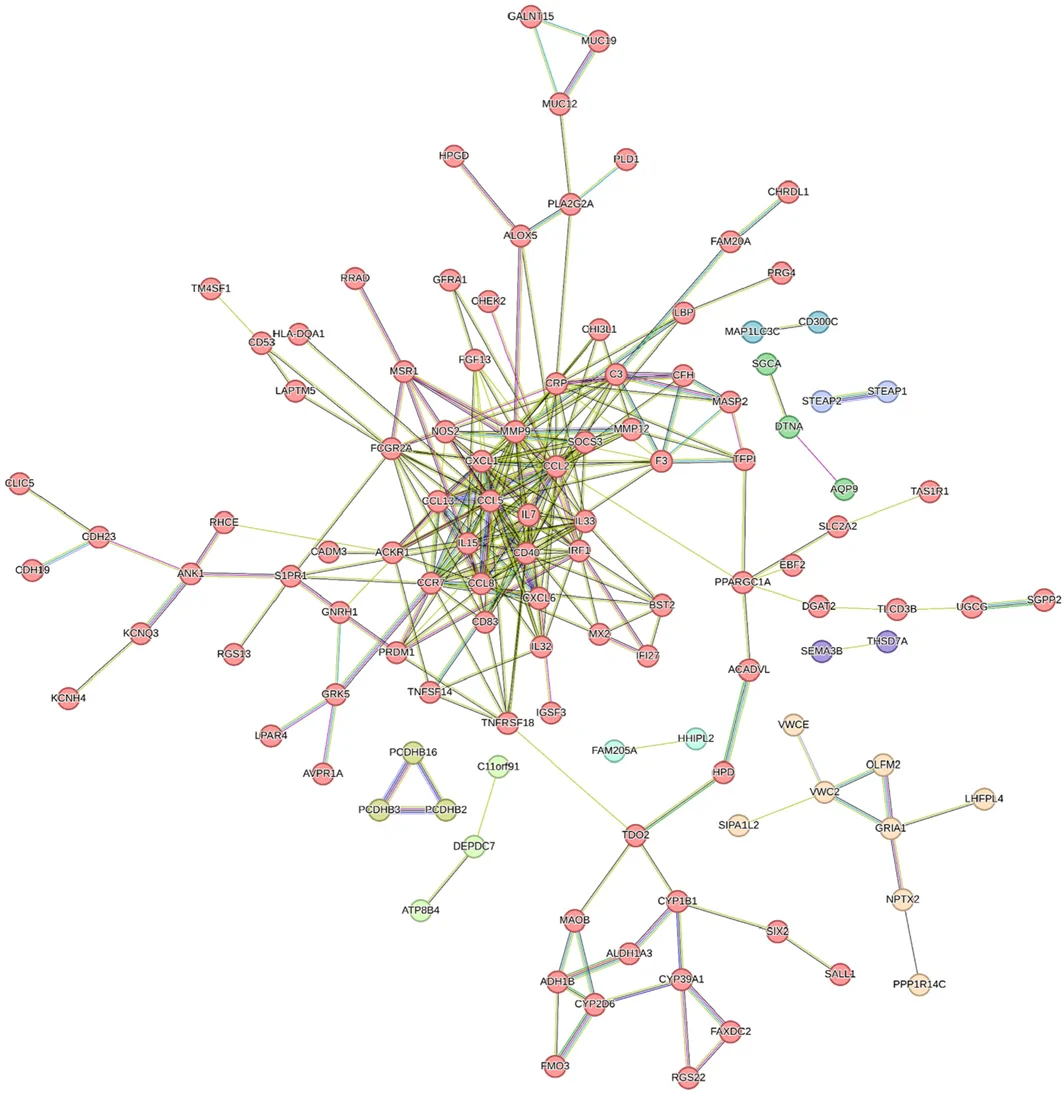
Protein–protein interaction (PPI) network and hub gene identification for upregulated transcripts. STRING interaction network of 300 upregulated DEGs in aged fibroblasts. K‐means clustering resolved a primary cluster of 90 interconnected nodes. CytoHubba Maximal Clique Centrality (MCC) analysis identified major hub genes including CCL5, CCL2, CXCL1, CD40, IL15, and MMP9. MCC, Maximal Clique Centrality.

Conversely, the PPI network for the 177 downregulated DEGs exhibited a more diffuse topology (Figure 10), with k‐means clustering resolving a core cluster of 29 genes (15% of downregulated DEGs). The highest‐scoring hub nodes in this metabolic cluster were SREBF1, FASN, SCD, and DHCR7. Enrichment analysis of this cluster highlighted lipid metabolism pathways, with the top‐scoring processes linked to SREBF1 being the positive regulation of cholesterol biosynthesis and the AMPK signaling pathway. Additional enriched pathways included fatty acid metabolism and the biosynthesis of unsaturated fatty acids, highlighting suppression of sterol and fatty acid biosynthetic pathways in aged fibroblasts.

**FIGURE 10:**
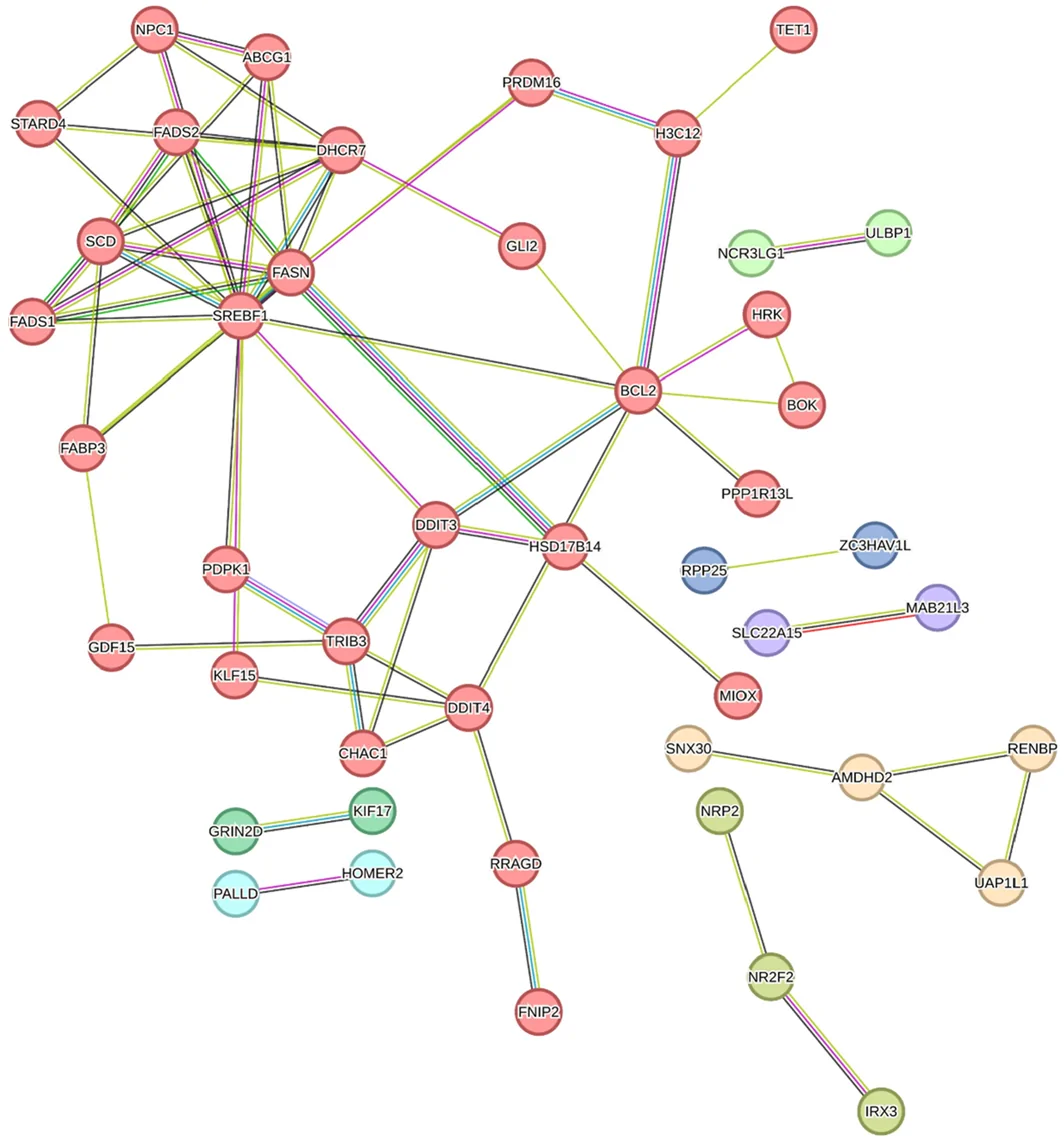
Protein–protein interaction (PPI) network of downregulated transcripts. STRING interaction network of 177 downregulated DEGs in aged fibroblasts. K‐means clustering identified a primary cluster of 29 nodes enriched in lipid metabolism, with top hub nodes SREBF1, FASN, SCD, and DHCR7.

## 4 DISCUSSION

Fibroblasts, like most mammalian cells, respond dynamically to their surrounding microenvironment to perform fundamental biological functions, including matrix synthesis, contraction, migration, and proliferation. Their functional state is regulated by both soluble biochemical cues and insoluble structural signals within the extracellular matrix. Previous studies have demonstrated that young fibroblasts transplanted into an artificially fragmented or degraded “aged” 3D collagen matrix acquire phenotypic features of aged fibroblasts, including reduced ECM synthesis, elevated matrix metalloproteinases, and increased pro‐inflammatory signaling.^11^ Conversely, aged dermal fibroblasts cultured within intact or newly synthesized ECM scaffolds can partially recover youthful functional attributes, such as restored collagen synthesis and reduced oxidative stress.^15^, ^16^, ^17^

These observations suggest that the fibroblast phenotype is highly responsive to ECM structure and composition.^32^ In this sense, fibroblasts may function as the “aging clock,”. While the ECM provides the structural context that records the timing of this process. Thus, the aged fibroblast phenotype observed in vivo represents an adaptive response to changes in the ECM.

These dynamic interactions suggest that the dermal fibroblast phenotype in vivo is heavily influenced by ECM microenvironmental structure. In this framework, reduced ECM structural gene expression observed in aged skin in vivo may represent a dynamic, adaptive response to altered mechanical tension and matrix fragmentation rather than an irreversible, cell‐autonomous loss of synthetic potential. In support of this concept, our experimental findings demonstrate that when primary fibroblasts from aged skin (>80 year old) are isolated from their native fragmented microenvironment and cultured under the same monolayer conditions for 6–8 days, they express core structural ECM genes including collagens, proteoglycans, and glycoproteins at levels comparable to young control (20–30 year old) fibroblasts. We moderate our conclusions to emphasize that while these data show comparable synthetic gene expression in vitro, direct in vivo‐to‐in vitro transition kinetics within individual donor fibroblasts were not measured.

In marked contrast to ECM structural genes, primary fibroblasts from aged donors maintained elevated expression of immune‐, cytokine‐, and chemokine‐related transcripts (including CCL5, CCL2, CXCL1, CD40, IL15, and MMP9) following primary culture under the same conditions. We frame this persistent pro‐inflammatory expression pattern as a persistent age‐associated transcriptional phenotype. While persistence over 6–8 days in primary culture demonstrates a durable cellular memory, we present cell autonomy as a potential interpretation or hypothesis rather than an established mechanism, recognizing that residual epigenetic modifications acquired during decades in the aged tissue environment could account for sustained expression in vitro.

Several previous transcriptomic studies evaluated fibroblast aging after extended passage, cryopreservation, or tissue explant outgrowth over several weeks.^33^, ^34^, ^35^, ^36^, ^37^ Extended culture duration can select for specific proliferative subpopulations or attenuate transient microenvironmental adaptations.^38^ By evaluating primary cultures at early passage (6–8 days post‐isolation), our study captures a crucial time window wherein persistent transcriptional memory coexists with microenvironmental adaptation.

Despite similar initial growth rates and comparable core ECM expression during early culture, fibroblasts from aged donors exhibited clear age‐related differences in primary culture. Specifically, aged donor fibroblasts displayed telomeres that were approximately 1000 base pairs (∼1 kb) shorter than those of young controls (12 vs. 13 kb; *p* = 0.0016), reflecting cumulative replicative history in vivo. Furthermore, when passaged long‐term (>100 days), aged fibroblasts exhibited premature growth deceleration, achieving a 20% lower final PDL (43 vs. 53 PDLs; *p* < 0.001).

Only during later passages (>11 passages) did fibroblasts from aged donors exhibit a reduced proliferative capacity and a lower terminal PDL compared with fibroblasts from young donors. Similar early growth observations have been reported previously.^39^ These findings contrast with studies that directly isolate fibroblasts from aged skin. For example, single‐cell RNA sequencing of fibroblasts obtained directly from aged dermis revealed reduced proliferative capacity and delayed G1/S cell‐cycle progression compared with fibroblasts from young donors.^40^

Before extended culture, however, fibroblasts from young and aged donors retained clear biological differences. One such difference was telomere length. Telomeres are nucleoprotein complexes containing specific repeating nucleic acid sequences that cap linear chromosomal ends with double‐stranded DNA, and end with a single‐stranded G‐rich sequence to protect chromosomes from degradation.^41^ During successive rounds of cell division, telomeres progressively shorten, and this shortening is thought to contribute to cellular aging.^42^

In our study, fibroblasts derived from aged donors and cultured for only 6–8 days (until 70% confluence) exhibited telomeres approximately 1 kilobase shorter than those from young donors. This observation is consistent with previous reports that dermal fibroblast telomeres shorten at approximately 11 base pairs per year.^43^ Interestingly, environmental stressors such as ultraviolet exposure do not appear to significantly accelerate telomere shortening in dermal fibroblasts, as fibroblasts derived from sun‐exposed and sun‐protected skin exhibit similar telomere lengths. Thus, despite measurable differences in telomere length, fibroblasts from young and aged donors grew similarly, under the same culture conditions.

The enrichment of cytokine signaling and pro‐inflammatory networks in aged fibroblasts aligns with the broader framework of “inflammaging”, the chronic, low‐grade systemic inflammation associated with advanced age.^44^, ^45^ In this model, aging is characterized by a progressive accumulation of cellular damage and reduced capacity to resolve physiological stress, leading to a sustained pro‐inflammatory environment. Numerous studies have since reported chronic, low‐level inflammation as a hallmark of aging across multiple tissues.^46^, ^47^, ^48^ A key limitation of the present study is that our conclusions regarding the pro‐inflammatory phenotype of primary fibroblast cultures from aged human skin rely primarily on bulk RNA sequencing and bioinformatic network analyses. Future studies incorporating independent RT‐qPCR validation and protein‐level quantification of secreted cytokines and chemokines will be important to confirm these cells' functional secretory output.

Potential epigenetic mechanisms such as altered DNA methylation or histone modifications may underpin the stability of this pro‐inflammatory transcriptional signature in aged dermal fibroblasts Wang et al.^49^ Although investigation of epigenetic regulation is beyond the scope of the present study, we highlight epigenetic regulation as an attractive hypothesis for persistent cellular memory in dermal fibroblast aging.

Our findings support the important concept that fibroblast involvement in inflammaging is not strictly cell‐autonomous, but operates through a reciprocal feed‐forward loop with the extracellular microenvironment. Persistent secretion of MMPs and inflammatory mediators by aged fibroblasts promotes localized matrix degradation, which in turn alters structural and mechanical cues, reinforcing the aged phenotype.^11^ Thus, dermal aging is better conceptualized as allostasis—a continuous adjustment of physiological set points in response to changing external conditions—rather than simple homeostasis.^38^ Whether newly identified single‐cell dermal fibroblast subclusters represent distinct, lineage‐committed subpopulations or highly adaptable functional states shaped by local microenvironmental niches remains an important direction for future investigation.^40^, ^50^

In conclusion, our study demonstrates a critical divergence in dermal fibroblast aging: while reduced ECM structural gene expression in aged skin reflects a flexible, microenvironment‐dependent adaptation, the pro‐inflammatory transcriptional phenotype persists in primary culture. Dissecting the interplay between microenvironmental cues and persistent cellular memory will advance our understanding of skin aging and inform therapeutic strategies to restore dermal tissue resilience.

## AUTHOR CONTRIBUTIONS

**Yilei Cui:** Conceptualization; methodology; data collection; interpretation; validation; formal analysis; writing—original draft; data presentation. **Mai Shi:** Data analysis. **Christal Worthen:** Conceptualization; methodology; data collection; formal analysis; validation. **Sherilyn Grill:** Data collection; formal analysis; validation; writing—review and editing. **Jayakrishnan Nandakumar:** Data analysis; validation; writing—review and editing. **Yi Zhao:** Writing—review and editing. **Gary J. Fisher:** Conceptualization; data analysis; interpretation; validation; writing—review and editing; data presentation; supervision; funding acquisition; resources.

## CONFLICT OF INTEREST STATEMENT

The authors declare no conflicts of interest.

## ETHICS STATEMENT

The University of Michigan Institutional Review Board approved human subjects' participation in this study, and all subjects provided written informed consent before inclusion. The study followed the principles of the Declaration of Helsinki.

## Data Availability

The data that support the findings of this study are available from the corresponding author upon reasonable request.
